# P-1724. Knowledge, Attitude, and Practices (KAP) on Appropriate Antibiotic Use among Healthcare Providers in Qatar - A cross-sectional study

**DOI:** 10.1093/ofid/ofae631.1888

**Published:** 2025-01-29

**Authors:** Sherin Shams, Hanaa Nafady-Hego, Asma Ali Al Nuaimi, Samah Saleem, Maryan Hassan Aziz, Naheel Ismail Seyam, Kamran Aziz, Dalia Kandil, Aimon Malik, Anil G Thomas, Mohamed Ghaith Al-Kuwari, Wael Ezzeldin Saeed, Samya Ahmad Al Abdulla, Jeyaram Illiayaraja Krishnan, Sandy Semaan, Abdul-Badi Abou-Samra, Adeel A Butt

**Affiliations:** Hamad Medical Corporation, Doha, Ad Dawhah, Qatar; Microbiology and immunology, Faculty of Medicine, Assiut University, Assiut, Egypt., Doha, Ad Dawhah, Qatar; Primary Health Care Corporation, Doha, Qatar, Doha, Ad Dawhah, Qatar; Hamad Medical corporation, Doha, Ad Dawhah, Qatar; Primary Health Care Corporation, Doha, Qatar, Doha, Ad Dawhah, Qatar; Primary Health Care Corporation, Doha, Qatar, Doha, Ad Dawhah, Qatar; Primary Health Care Corporation, Doha, Qatar, Doha, Ad Dawhah, Qatar; Primary Health Care Corporation, Doha, Qatar, Doha, Ad Dawhah, Qatar; Hamad Medical corporation, Doha, Ad Dawhah, Qatar; Hamad Medical corporation, Doha, Ad Dawhah, Qatar; Primary Health Care Corporation, Doha, Ad Dawhah, Qatar; Primary Health Care Corporation, Doha, Qatar, Doha, Ad Dawhah, Qatar; Primary Health Care Corporation, Doha, Qatar, Doha, Ad Dawhah, Qatar; Primary Health Care Corporation, Doha, Qatar, Doha, Ad Dawhah, Qatar; Primary Health Care Corporation, Doha, Qatar, Doha, Ad Dawhah, Qatar; Hamad Medical corporation, Doha, Ad Dawhah, Qatar; Weill Cornell Medicine, Doha, Ad Dawhah, Qatar

## Abstract

**Background:**

There has been growing awareness of antibiotic resistance as a significant public health issue, notably a widespread prescription of antibiotics for upper respiratory tract infections (URTIs).

Understanding the knowledge, attitude, and practices (KAP) of healthcare providers is crucial in designing interventions to reduce inappropriate antibiotics use. Our aim was to determine the KAP of primary care physicians towards antibiotics prescriptions in the outpatient setting to develop strategies to improve appropriate antibiotics use.

Physician responses to KAP Survey.

**Figure:**
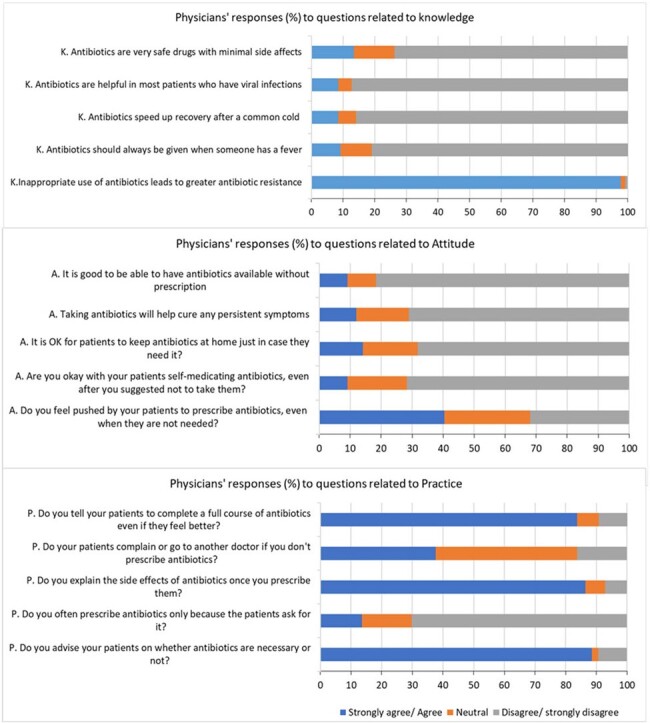

**Methods:**

We employed a cross-sectional survey to determine the KAP of physicians at 4 Primary Healthcare Centers (PHCCs) in Qatar. The KAP questionnaire was structured into three main sections: evaluating physicians' knowledge regarding Upper Respiratory Infections (URIs) and antibiotics, examining attitudes encompassing beliefs or perspectives on antibiotic usage for URIs, and investigating practices to understand how physicians implement their knowledge and attitudes in their actions. A 5-point Likert scale was used for the questions.

**Results:**

A total of 141 physicians were surveyed and 96% responded. Nearly 80% were >40 years old, approximately half were women and 69% had ≥10 years of clinical experience. A majority of physicians (97.8%) agreed that inappropriate antibiotic use contributes to the rise of antibiotic resistance, 80.8% expressed disagreement with prescribing antibiotics for fever alone, and 40.4% admitted to experiencing patient pressure to prescribe antibiotics even when unnecessary. Conversely, 13.4% admitted to frequently prescribing antibiotics solely due to patient requests, and 37.6% confirmed encountering patient complaints or patient migration to other doctors if antibiotics were not prescribed.

**Conclusion:**

Primary care physicians are knowledgeable about the role and risks associated with antibiotics, but frequently felt pressured by the patients/families to prescribe antibiotics. Intervention strategies to educate the public and empower the physicians are needed to address the inappropriate use of antibiotics.

**Disclosures:**

**Adeel A. Butt, MD, MS**, Gilead Sciences: Grant/Research Support

